# The Protective Effects of Endogenous PACAP in Oxygen-Induced Retinopathy

**DOI:** 10.1007/s12031-021-01846-2

**Published:** 2021-04-24

**Authors:** Timea Kvarik, Dora Reglodi, Dora Werling, Alexandra Vaczy, Petra Kovari, Edina Szabo, Krisztina Kovacs, Hitoshi Hashimoto, Tibor Ertl, Judit Gyarmati, Tamas Atlasz

**Affiliations:** 1 Department of Anatomy, MTA-PTE PACAP Research Team, Medical School, University of Pecs, Pecs, Hungary; 2 Department of Obstetrics and Gynecology, Medical School, University of Pecs, Pecs, Hungary; 3 Department of Biochemistry and Medical Chemistry, Medical School, University of Pecs, Pecs, Hungary; 4 Laboratory of Molecular Neuropharmacology, Graduate School of Pharmaceutical Sciences, Osaka University, Suita, Osaka Japan; 5 Department of Sportbiology, University of Pecs, Pecs, Hungary

**Keywords:** Retina, PACAP, Knock out, ROP, Neuroprotection

## Abstract

Pituitary adenylate cyclase–activating polypeptide (PACAP) is a neuropeptide having trophic and protective functions in neural tissues, including the retina. Previously, we have shown that intravitreal PACAP administration can maintain retinal structure in the animal model of retinopathy of prematurity (ROP). The purpose of this study is to examine the development of ROP in PACAP-deficient and wild-type mice to reveal the function of endogenous PACAP. Wild-type and PACAP-knockout (KO) mouse pups at postnatal day (PD) 7 were maintained at 75% oxygen for 5 consecutive days then returned to room air on PD12 to develop oxygen-induced retinopathy (OIR). On PD15, animals underwent electroretinography (ERG) to assess visual function. On PD16, eyes were harvested for either immunohistochemistry to determine the percentage of the central avascular retinal area or molecular analysis to assess angiogenesis proteins by array kit and anti-apoptotic protein kinase B (Akt) change by western blot. Retinas of PACAP-deficient OIR mice showed a greater central avascular area than that of the wild types. ERG revealed significantly decreased b-wave amplitude in PACAP KO compared to their controls. Several angiogenic proteins were upregulated due to OIR, and 11 different proteins markedly increased in PACAP-deficient mice, whereas western blot analysis revealed a reduction in Akt phosphorylation, suggesting an advanced cell death in the lack of PACAP. This is the first study to examine the endogenous effect of PACAP in the OIR model. Previously, we have shown the beneficial effect of exogenous local PACAP treatment in the rat OIR model. Together with the present findings, we suggest that PACAP could be a novel retinoprotective agent in ROP.

## Introduction

Premature birth may come together with diseases that compromise future life quality, such as retinopathy of prematurity (ROP). Preterm infants with extreme retinal immaturity and incompletely vascularized retina are exposed to alternating oxygen concentration, which initiates new, abnormal vessel formation. These vulnerable vessels can grow into the vitreous cavity, causing bleeding and retinal detachment in the worst cases. Despite advanced neonatal care and current therapeutic strategies, ROP still remains the leading cause of preventable childhood visual impairment. Therefore, there is a need for developing new therapeutic agents with the help of the widely used oxygen-induced ischemic retinopathy (OIR) animal models.

Pituitary adenylate cyclase–activating polypeptide (PACAP) is a 38-amino acid pleiotropic peptide known to act as a neurotransmitter, neuromodulator, and neurotrophic factor (Nakamachi et al. 2011; Ciranna and Costa 2019; Johnson et al. 2020; Gargiulo et al. 2020). PACAP consistently exerts protective effects in the nervous system and peripheral organs by mediating various physiological processes (Martinez-Rojas et al. 2021; Nonaka et al. 2020; Toth et al. 2020). The peptide is involved, among others, in autophagy through activation of MAPK/ERK signaling cascade (D'Amico et al. 2020), influences cell differentiation through various trophic and angiogenic factors (Maugeri et al. 2018), regulates cell migration (Maugeri et al. 2016), enhances motor neuron viability (Bonaventura et al. 2018), and shows antiproliferative effects in glioma cells (D’Amico et al. 2013).

The protective role of PACAP can also be observed in the retina against hypoxic, mechanical, and chemical injuries as reviewed by Nakamachi and colleagues and by our group (Atlasz et al. 2016; Nakamachi et al. 2012; D’Amico et al. 2021). PACAP is neuroprotective also in diabetic retinopathy (D’Amico et al. 2021). We have recently shown the potentially positive effect of intravitreally given PACAP injection on the vascular changes in the rat OIR model (Kvarik et al. 2016).

The role of endogenous PACAP has been explored with the help of PACAP gene-deficient mice. These mice show several developmental and behavioral alterations, including altered neurobehavioral, bone, and tooth development (Farkas et al. 2017; Fulop et al. 2019b; Jozsa et al. 2018), paradoxical age-dependent stress behavior (Biran et al. 2020), and altered light responses (Riedel et al. 2020). The strong neuro- and general cytoprotective role of PACAP are mainly due to its anti-apoptotic, antioxidant, and anti-inflammatory effects (Soles-Tarres et al. 2020; Toth et al. 2020). Due to the lack of these protective actions in PACAP-deficient animals, increased vulnerability and pathological responses have been observed in different peripheral injuries, such as kidney ischemia, callus formation, and cardiomyopathy (Jozsa et al. 2019; Mori et al. 2010; Reglodi et al. 2012) and also in injuries of the nervous system, such as spinal cord injury, ischemia, and nerve degeneration (Armstrong et al. 2008; Maugeri et al. 2020; Ohtaki et al. 2006; Tsuchikawa et al. 2012). This endogenous protective effect of the peptide has also been confirmed in carotid artery occlusion-induced retinal injury (Szabadfi et al. 2012). It was found that PACAP gene-deficient mice reacted with a higher degree of retinal cell loss and reduction of the retinal layers than their wild-type mates. This could be counteracted by exogenous PACAP treatment (Szabadfi et al. 2012). Similarly, increased sensitivity has been observed in retinal inflammation induced by endotoxin (Vaczy et al. 2018). Levels of protective factors were decreased to a greater extent, while inflammatory cytokine increase was more intense along with a marker Muller glial cell activation in PACAP knockout (PACAP KO) mice (Vaczy et al. 2018). As the protective effects of PACAP are linked with aging processes, it is not surprising that PACAP-deficient mice also show accelerated aging (Reglodi et al. 2018a). Accelerated systemic amyloidosis and increased levels of oxidative stress markers have been found in PACAP KO mice (Ohtaki et al. 2010; Reglodi et al. 2018b). In the eye, age-related loss of lacrimal gland function and accelerated retinal aging have been described (Kovacs-Valasek et al. 2017; Nakamachi et al. 2016). These results clearly show that endogenously present PACAP reacts as a stress-response peptide necessary for protection against different retinal insults. The aim of the present study was to investigate whether PACAP exerts similar protective effects endogenously also in a model of retinopathy of prematurity. Our observation was that retinopathic mice lacking PACAP showed a deteriorated vascularization, a disrupted cytokine balance, and a decreased cell protective mechanism, as well as visual functional disturbances. These results suggest that endogenous PACAP is part of the protective machinery in this retinopathy model.

## Materials and Methods

### Animals

Mice were obtained from breeding colonies maintained at the Animal Facility of Medical School, University of Pecs (Pecs, Hungary). Wild-type and homozygous PACAP-deficient mice were used. PACAP-deficient mice were generated and maintained on CD1 background as previously described (Hashimoto et al. 2001, 2009); they were backcrossed for ten generations with the CD1 strain. Each nursing mum with their litters was housed in individual cages, fed, and watered ad libitum under 12/12-h light/dark cycles. Animal housing, care, and application of experimental procedures were in accordance with the ethical guidelines approved by the University of Pecs (BA02/2000-31/2011) and directives of the National Ethical Council for Animal Research, the European Communities Council (86/609/EEC), and ARVO Statement for the Use of Animals in Ophthalmic and Vision Research.

### Experimental Design

To induce retinopathy, wild-type (OIR-Wt, *n *= 15) and PACAP-deficient (OIR-KO, *n* = 14) pups with their nursing mother were kept in an oxygen chamber (Biospherix Ltd., NY, USA) supplied by an oxygen sensor (ProOx 110, Biospherix Ltd., NY, USA) to monitor and maintain continuous 75% of oxygen concentration from PD7 to PD12. Then, they were returned to room air until PD16. Control litters of both genotypes (Cont-Wt, *n* = 15, Cont-KO, *n *= 15) were room-air reared during the whole experiment.

### Electroretinography

Some of the animals from each group (Cont-Wt, *n* = 4, Cont-KO, *n* = 3, OIR-Wt, *n* = 3, OIR-KO, *n* = 4) underwent electroretinography (ERG) examinations to assess visual function on PD15 after an overnight dark adaptation. The measurements were performed as previously described by Danyadi and co-workers (Danyadi et al. 2014). Briefly, after systemic anesthesia, the pupils were dilated with 0.5% cyclopentolate (Humapent-Teva, TEVA Ltd., Hungary) and topically anesthetized with Oxybuprocaine 0.4% (Humacain-Teva, TEVA Ltd., Hungary) eye drops. ERGs were recorded by surface electrodes from the center of the cornea with a negative electrode placed subcutaneously between the eyes and a ground electrode inserted under the skin of the back. The responses to light flashes (5.0 cd/m^2^, 0.25 Hz, 503 nm green LED light) were pre-amplified, amplified, and recorded by an A/D converter (Ratsoft-Solar Electronic). Responses were averaged with the software of the A/D converter. The selected parameters were measured by OriginPro 2016 software (OriginLab Corporation, MA, USA) and statistically analyzed by ANOVA with Fisher’s post hoc test after test for homogeneity of variance (STATISTICA, StatSoft Inc., OK, USA).

### Immunohistochemistry

Isolectin immunohistochemical staining was performed as previously described by Connor and co-workers (Connor et al. 2009). Briefly, after anesthesia on P16 ± 1, eyes were removed and immediately placed into 4% PFA for fixation at room temperature. After 1 h, eyes were washed three times in PBS, and retinas were isolated under a dissecting microscope. To stain the retinal vasculature, 500 µl fluoresceinated isolectin solution (Isolectin GS-IB4 from Griffonia simplicifolia, Alexa Fluor 568 conjugate; Thermo Fischer Scientific Inc., MA, USA) was added to the isolated retinas. After an overnight rocking in lectin solution at room temperature, retinas were rinsed three times in PBS. Finally, four incisions were made to flatten the retinas onto microscope slides and were covered with a coverslip with mounting media (Fluoromount, Sigma-Aldrich Co., MO, USA). Digital photographs were taken with a Nikon Eclipse 80i fluorescence microscope (Nikon, Melville, NY, USA).

### Assessment of Vessel Morphology

A trained observer blinded for the groups evaluated the ratio of central avascular retinal territory, and vessel density. Measurements were made by Adobe Photoshop CS6 (Adobe Systems Inc., CA, USA) and ImageJ software (National Institutes of Health, Bethesda, MD, USA). The central avascular retina was outlined and measured, and its percentage to the whole retina was given. Vessel density was calculated by marking out all the vessels in the intact retina and giving their portion to the whole vascularized territory.

Results are represented in mean ± SEM. Statistical analysis was performed by independent t-test after Levene’s *F*-test for equality of variance using STATISTICA software (StatSoft Inc., OK, USA).

### Angiogenesis Array Analysis

After euthanasia on PD 17 ± 1, the eyeballs were removed, and retinas were carefully separated and quickly frozen in dry ice. Angiogenesis proteins were investigated from pooled tissue homogenates by semiquantitative Mouse Angiogenesis Array Kits (R&D Systems, Bio-Techne Ltd., MN, USA). In these arrays, the sample proteins bind to selected captured antibodies spotted on nitrocellulose membranes. The kits contain all buffers, detection antibodies, and membranes necessary for the measurement. The arrays were performed as described by the manufacturer’s protocol. In brief, after blocking the membranes for 1 h and adding the detection antibody cocktail for another 1 h at room temperature, the membranes were incubated with 1.5 ml tissue homogenates at 2–8 °C overnight on a rocking platform. After washing with buffer three times, membranes were incubated with horseradish peroxidase–conjugated Streptavidin at room temperature and exposed to chemiluminescence detection reagent to develop X-ray films. The arrays were repeated two times. For data analysis, films were scanned and mean pixel densities of interested proteins, selected by eye control, were measured by ImageJ software and were normalized to the reference spots. To compare the possible differences between angiogenetic profiles of the different groups, we determined the relative density change of the selected spots. Only those proteins are represented, which showed at least a 1.3-fold change.

### Western Blot Analysis

For western blot experiments, tissue homogenates of four retinas per group were used. Frozen tissues were homogenized with the Ultra-Turrax and Potter homogenizer in 150 μl lysis buffer (50 mM Tris, 50 mM EDTA, 0.5% protease inhibitor cocktail (Sigma-Aldrich), and 0.5% phosphatase inhibitor cocktail (Sigma-Aldrich), pH = 7.4). The homogenate was sonicated, and the protein concentration was determined with a DC™ Protein Assay kit (Bio-Rad) according to the manufacturer’s description. The tissue lysate was diluted in Laemmli buffer, boiled for 5 min, centrifuged (13,300 rpm, 10 min), and the clear supernatant was used for further investigations. Tissue extracts were separated with SDS-PAGE with protein loads of 20 μg/lane and transferred onto a nitrocellulose membrane. The membranes were blocked with 5% non-fat dried-milk proteins in Tris-buffered saline (TBS) and 0.1% Tween, incubated with anti-Akt (No. 9272), anti-Akt1 Ser473 (No. 9271) antibody (both from Cell Signaling Technology) at 4 °C overnight at a dilution of 1:1000. The secondary antibody was horseradish peroxidase–conjugated goat anti-rabbit IgG. Peroxidase labeling was visualized with the Pierce ECL Western Blotting Substrate (Thermo Scientific) detection system. Quantification of band intensities of the blots was performed by ImageJ software. Pixel volumes of the spot were normalized to the internal controls. Data are represented by pixel density in arbitrary units. Statistical analysis was performed by ANOVA test with Fisher’s post hoc analysis after test for homogeneity of variance using STATISTICA software (StatSoft Inc., OK, USA).

## Results

### Vessel Morphology

**Fig. 1:**
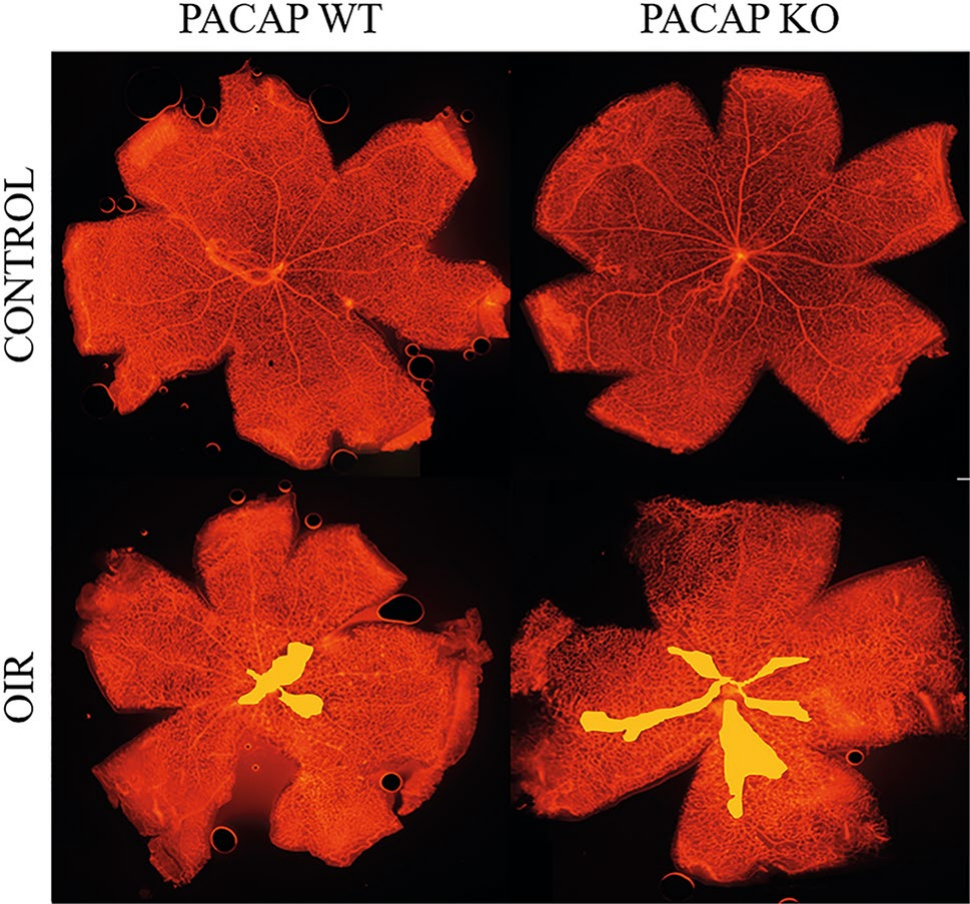
Representative pictures of mouse retinas to visualize retinal vasculature. The presence of oxygen-induced retinopathy (OIR) is indicated with a yellow area. PACAP deficiency affects the extent of retinopathy; control animals have completely vascularized retina on PD16

**Fig. 2:**
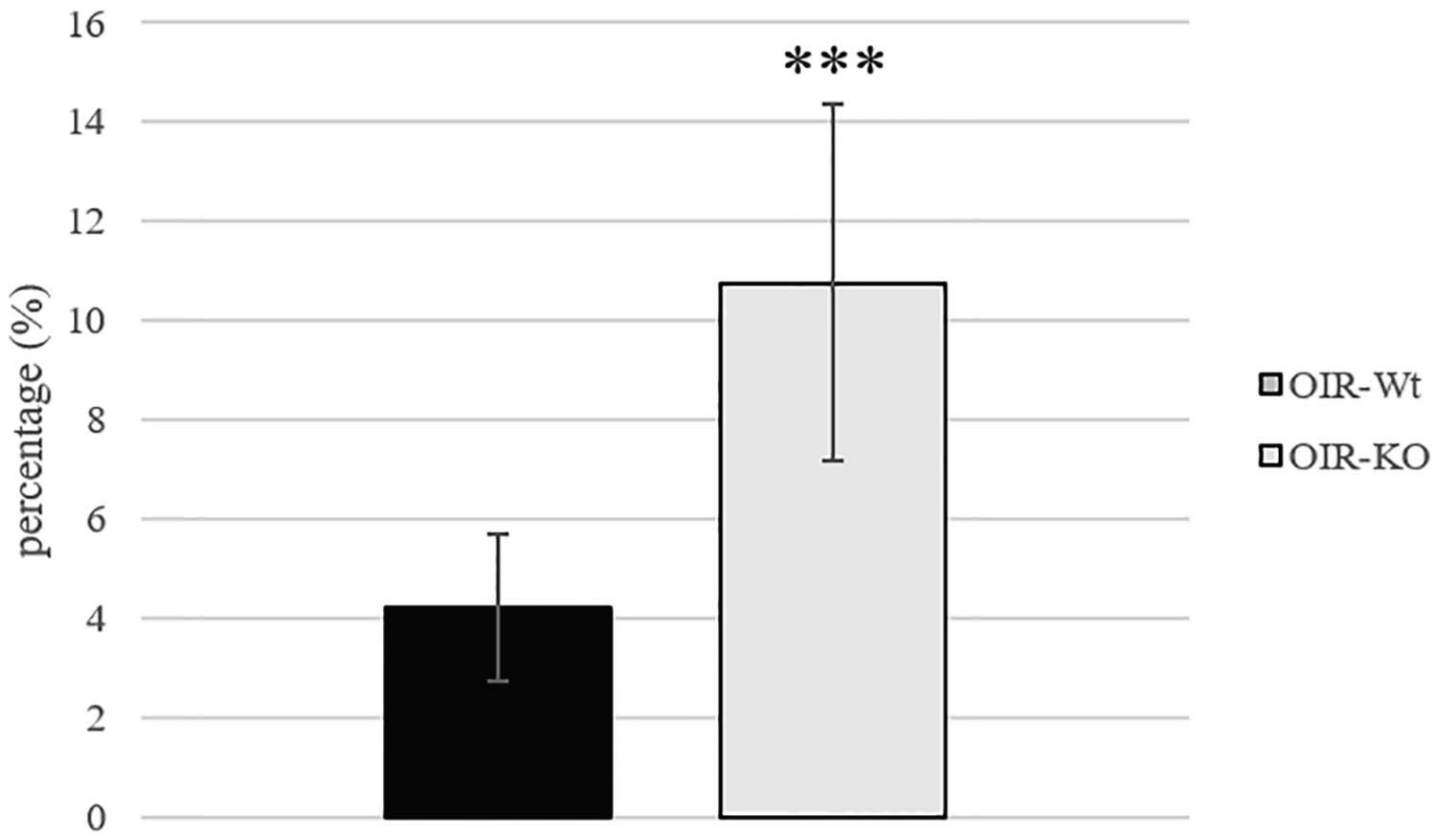
Percentage (%) of the avascular retinal area of mice with retinopathy is represented as mean ± SEM. PACAP-deficient mice developed a bigger central avascular retinal area. *** *p* < 0.000001

On PD16, the retinal vasculature of room-air raised pups of PACAP wild-type and KO groups reached the ora serrata, and no avascular area or abnormal vessel formation was observed (Fig. 1). In OIR groups, the avascular area was formed in the center of the retina. The ratio of the avascular territory to the whole retinal area was 10.77 ± 0.16% in OIR-KO mice and 4.22 ± 0.37% in OIR-Wt mice, which was a significant difference (*p* < 0.000001) (Fig. 2). No difference was found in vessel density between OIR-Wt and OIR-KO mice (27.31 ± 2.77 vs. 29.77 ± 3.46% respectively, *p* = 0.59).

### Electroretinography

**Fig. 3:**
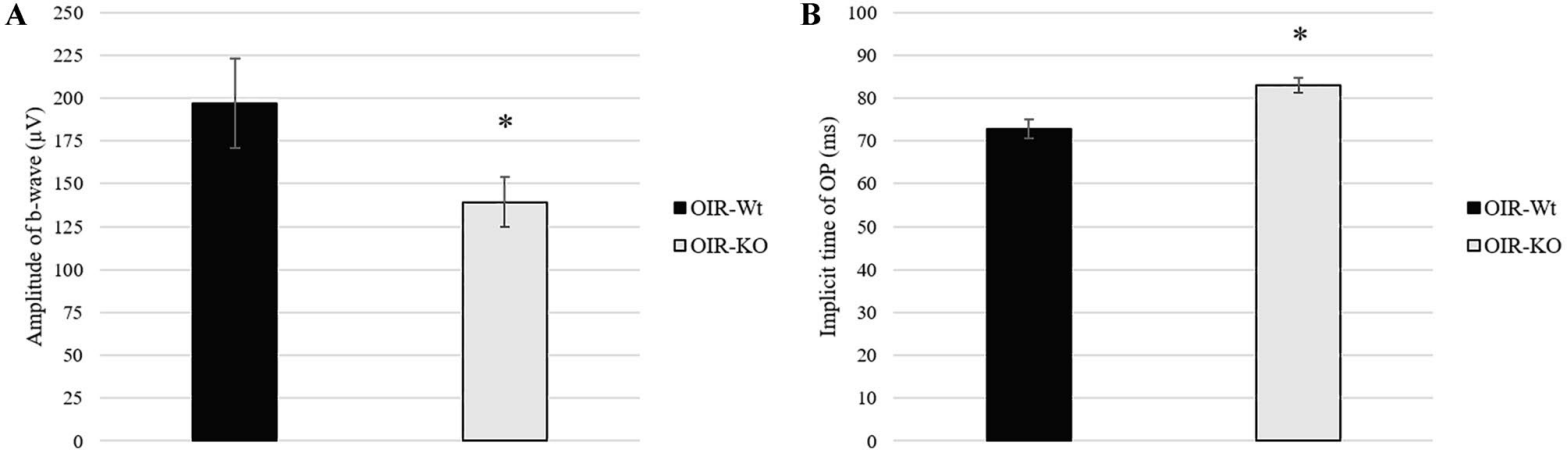
Measurement of electroretinography amplitudes in PACAP wild-type (Wt) and knockout (KO) mice with oxygen-induced retinopathy (OIR). Amplitudes of b-wave **A** and averaged oscillatory potential **B** were measured on PD15 after overnight dark adaptation. Values are expressed as mean ± SEM, *p < 0.05

The visual function of mice was assessed by ERG examination after an overnight dark adaptation on PD15. The waves were not significantly different between control and OIR wild-type groups. Amplitudes of b-waves demonstrating the depolarization of Muller cells were significantly decreased in the OIR-KO group compared to the OIR-Wt group (Fig. 3A). Average oscillatory potential amplitudes of OIR-KO mice were higher than those of the OIR-Wt (Fig. 3B).

### Angiogenesis Array

**Fig. 4:**
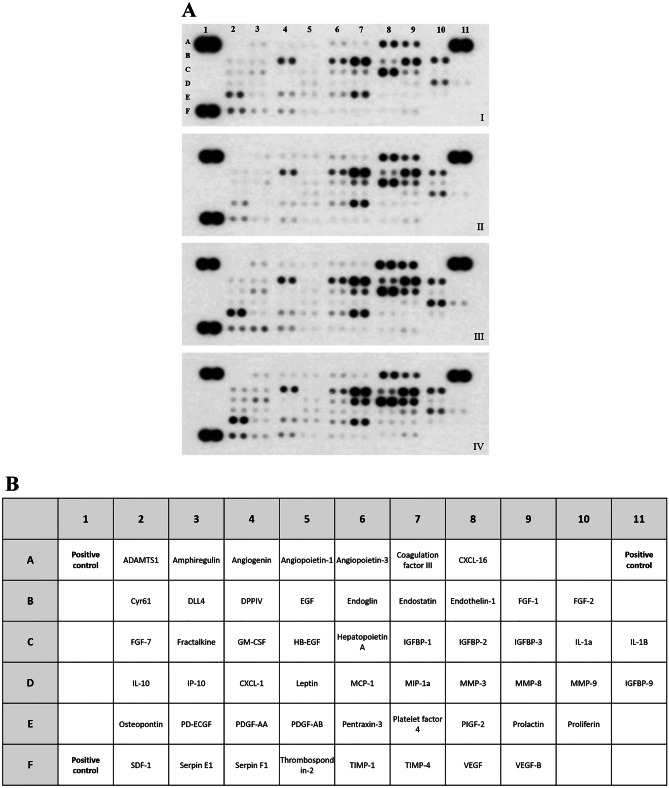
**A** Representative panels show cytokine arrays from homogenates of control (I), control-PACAP-deficient (II), wild type OIR (III), and PACAP-deficient oxygen-induced retinopathy (OIR) retinas (IV). **B** The table indicates the examined cytokines in each box

**Fig. 5:**
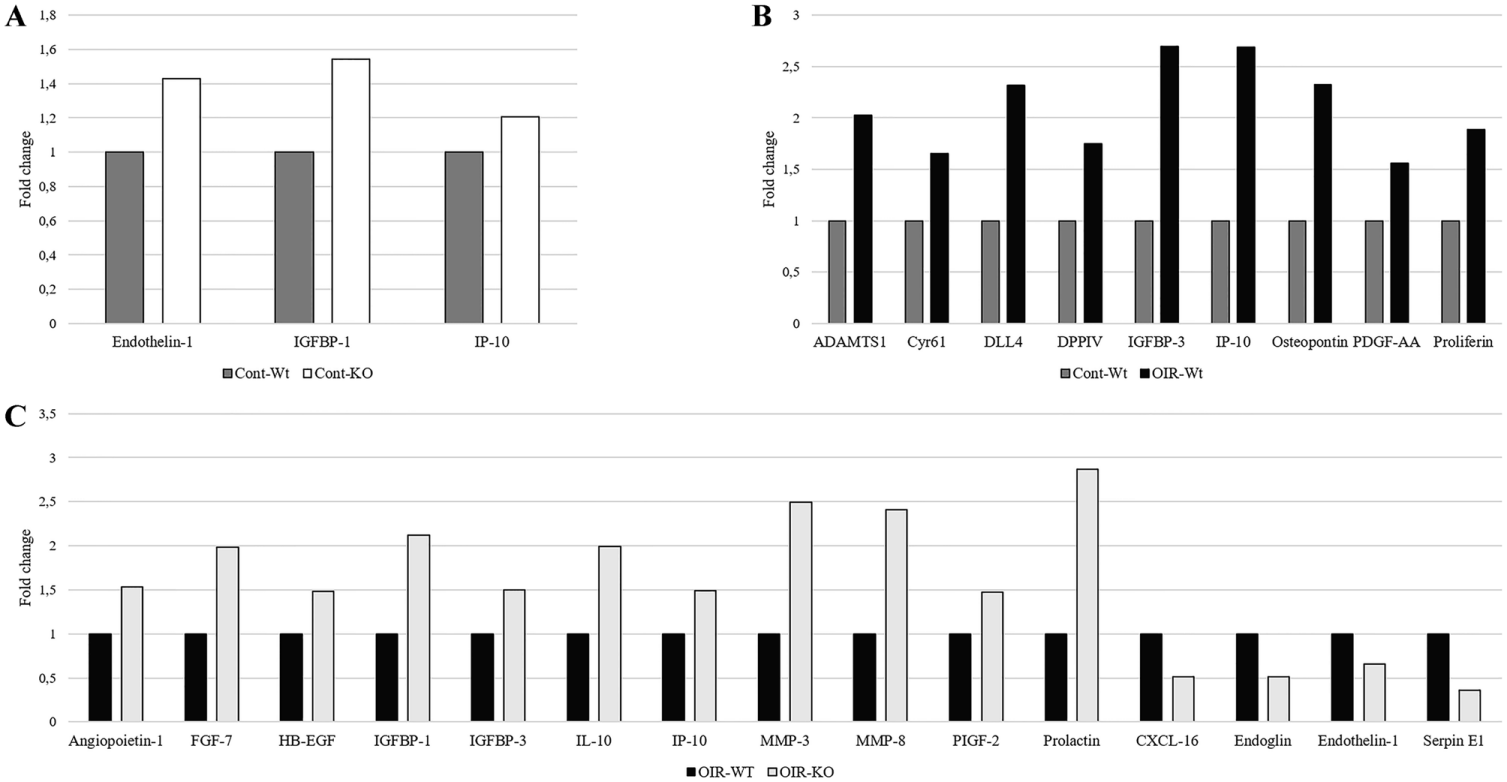
Semiquantitative analysis of angiogenesis related proteins. The pro- and anti-angiogenic factors whose expressions in the retina of control PACAP-deficient **A** or wild-type oxygen-induced retinopathy (OIR) **B** or PACAP-deficient OIR mice **C** showed more than 30% relative change compared to either control wild-type or OIR wild-type mice are represented in bar charts. The results are based on two independent measurements

Altogether 53 angiogenesis-related proteins were tested using a semiquantitative angiogenesis array (Fig. 4A, B). As result of OIR, 9 angiogenic factors (i.e., CYR61, ADAMTS-1, IP-10, Osteopontin, Proliferin) were upregulated in wild-type mice (Fig. 5B). In the retina of PACAP-deficient mice with retinopathy, we observed an increase in altogether 11 more factors, including proteins related to extracellular matrix reorganization (i.e., MMP-3, MMP-8), inflammation (i.e., IL-10, IP-10), and growth factors (i.e., FGF-7, IGFBP-1, PIGF-2), and the decrease of 4 proteins, such as CXCL-16, Endoglin, Endothelin-1, and Serpin E1, also known as Plasminogen activator inhibitor-1 (PAI-1) (Fig. 5C). Under control circumstances, few proteins (Endothelin-1, IGFBP-1, IP-10) showed alteration in KO animals (Fig. 5A).

### Western Blot Analysis

**Fig. 6:**
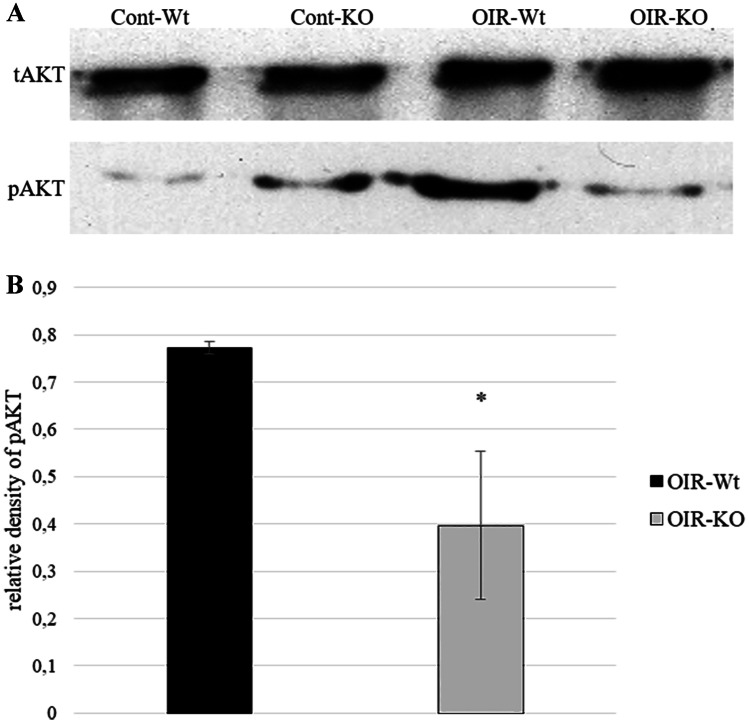
Activation of AKT was determined in mouse retinas on PD16. Total proteins (non-phosphorylated) were used as loading controls. Representative blots **A** and bar chart **B** of the quantified blots are presented. Bars represent mean ± SEM of pixel densities; * indicates significant difference between PACAP knockout oxygen-induced retinopathy (OIR-KO) and wild-type (OIR-Wt) mice (*p* = 0.027)

The phosphorylation level of anti-apoptotic Akt did not change in control KO mice. A strong Akt activation was detected in wild-type retinopathic animals (OIR-Wt), whereas results of retinopathic KO mice (OIR-KO) showed a significant decrease in Akt phosphorylation (Fig. 6A, B).

## Discussion

Improved perinatal care leads to an increased survival rate of very low birth weight preterm infants. These premature babies are at risk of developing retinopathy of prematurity, a disease leading to vision impairment or eye problems with various degrees. The estimated worldwide ROP incidence among babies weighing less than 1500 g is approximately 30% (Cavallaro et al. 2014). The current therapeutic options might halt the progress of the disease in mild cases, but the real cure for this condition still awaits to be discovered. Animal models can mimic the development and characteristics of ROP, so they help to study potential retinoprotective agents, such as PACAP. Its strong neuroprotective and neurotrophic effects have been well described in several retinal pathologies. Exogenously applied PACAP attenuates retinal excitotoxic injury caused by monosodium glutamate (Atlasz et al. 2009; Tamas et al. 2004), ischemic retinal lesion (Atlasz et al. 2007; 2010), and UV-light induced retinal degeneration (Atlasz et al. 2011). Previously, we have shown that three times intravitreal PACAP administration during the first 2 weeks of life ameliorates the retinopathy seen in the ROP model of neonatal rats (Kvarik et al. 2016).

In the present study, we used an established mouse model of retinopathy of prematurity to evaluate the effect of lacking endogenous PACAP with the help of PACAP-deficient mice. Here, we first showed that retinal flat mounts of KO mice demonstrated a significant increase of central avascular area on PD 16, suggesting that PACAP deficiency leads to a more severe form of retinopathy. This assumption is further confirmed by functional electroretinography examination where the b-wave amplitudes—reflecting the activity of bipolar and third-order retinal cells—are decreased in the retinopathic PACAP-deficient mice.

Several studies have described that under physiological conditions, there is no remarkable difference in the gross morphology of PACAP KO mice compared to wild types (Kovács-Valasek et al. 2017; Szabadfi et al. 2012; Vaudry et al. 2005). However, slight biochemical, synaptic, and ultrastructural alterations were observed in the nervous system regarding axonal arborization, myelination process, inner ear structure, and cerebellar migration (Allais et al. 2007; Tamas et al. 2012; Vincze et al. 2011; Yamada et al. 2010). Data obtained from PACAP-deficient mice provide evidence that lack of the neuropeptide leads to an increased vulnerability to stressors. PACAP KO mice exhibited a more severe pathology of experimental autoimmune encephalomyelitis, delayed axonal regeneration in peripheral nerve injury, and increased infarct size in cerebral ischemia (Armstrong et al. 2008; Chen et al. 2006; Nakamachi et al. 2010; Tan et al. 2009). Recent studies have shown that PACAP KO animals display early-onset and a more generalized form of systemic amyloid deposition throughout the body in various organs (Reglodi et al. 2018b), and PACAP deficiency makes the articular cartilage structure more prone to degenerate (Szegeczki et al. 2019; Lauretta et al. 2020). PACAP-deficient mice also display accelerated hearing impairment compared to wild-type mice (Fulop et al. 2019a). The protective effect of endogenous PACAP has also been described in retinal toxic, ischemic, metabolic, and inflammatory lesions (Kawaguchi et al. 2010; Szabadfi et al. 2012; Vaczy et al. 2018). Endo and colleagues have demonstrated that even the partial lack of endogenous PACAP leads to the aggravation of the toxic neuronal damage. They have shown that after 7 days of intravitreal NMDA injection, the number of retinal ganglionic cells decreased significantly in PACAP heterozygous mice relative to their wild-type counterparts (Endo et al. 2011). In the mouse model of transient retinal ischemia, Szabadfi et al. has reported that all retinal layers suffered more severe damage after a 10-min ischemia followed by a 2-week reperfusion period in PACAP-deficient mice than in wild-type mice (Szabadfi et al. 2012). Recently, Vaczy and co-workers have revealed that intraperitoneal injection of lipopolysaccharide (LPS) led to a markedly more severe eye inflammation in PACAP KO mice with a decrease in anti-apoptotic protein kinase B (pAkt) level and a more expressed elevation of sICAM-1, JE, TIMP-1 cytokines than in wild-type mice (Vaczy et al. 2018). The changes in the protective protein profile are in accordance with the generally observed PACAP-induced proteomic and transcriptomic changes in neuronal injuries (Rivnyak et al. 2018). Moreover, the use of PACAP and PACAP derivative eye drops in the ischemic retinopathy model was found to be retinoprotective as it passes the ocular barriers (Atlasz et al. 2019; Werling et al. 2016, 2017). These results are in accordance with our observations that under normoxic conditions, there is no gross morphological aberration in retinal vessel development, but after a 5-day hyperoxic insult, PACAP deficient mice had a significantly greater avascular central retinal territory than their wild-type littermates.

The mechanism of the protective effect of PACAP has been studied extensively, mainly in the nervous system and in the retina. It has been revealed that PACAP influences anti-apoptotic pathways, like ERK, CREB, Bcl-2, Bcl-xl, and Akt, while it inhibits pro-apoptotic proteins, such as caspases and Bad in glutamate-induced retinal injury in neonatal rats (Racz et al. 2006, 2007). In a rat retinal hypoperfusion model, the MAPKs and Akt signaling pathways were studied after PACAP injection. It was shown that PACAP treatment led to a marked significant increase in the level of phosphorylated Akt (Szabo et al. 2012). It was also shown that LY294002, a PI3K inhibitor, can inhibit retinal neovascularization via downregulation of the PI3K/AKT-VEGF pathway (Di and Chen 2018). The same observations have been reported in connection with excitotoxic retinal injury and other ischemic/reperfusion lesions (May et al. 2010; Racz et al. 2008). The anti-apoptotic action of endogenous PACAP through Akt signaling has been further strengthened by Vaczy et al. in their retinal inflammation model. They have reported a decrease in pAkt levels in LPS-injected PACAP KO mice compared to wild-type mice (Vaczy et al. 2018). Our western blot results correlate with these findings. We observed a robust increase in the phosphorylation state of Akt in wild-type retinopathic mice, while it markedly decreased in PACAP-deficient retinopathic mice. This indicates a disturbance in protective mechanisms against apoptosis, leading to an increased vulnerability in the case of PACAP deficiency.

These data show that endogenous PACAP is protective in a model of retinopathy which is mainly related to the disturbance of retinal vascularization. Diabetic retinopathy and ROP share common features in their progression. Hypoxic circumstances in the retinal tissue switch the chain of reaction leading to new, abnormal vessel formation. The critical factors in this process are the members of the hypoxia-inducible factor family (HIFs) and a consequential VEGF expression. The protective effect of PACAP in diabetic retinopathy and diabetic macular edema (DME) has been demonstrated in various studies. A single dose of intravitreal PACAP injection reduced the expression of inflammatory cytokine Il-1B and downregulated VEGF and its receptors in the diabetic rat model (D’Amico et al. 2017b). The disruption of tight junctions in the outer blood retinal barrier (BRB) generates the progress of DME. Scuderi and colleagues suggested that PACAP can maintain the integrity of outer BRB as it was able to counteract the cell junction protein damage in ARPE-19 cells cultured in a hyperglycemic and inflammatory milieu (Scuderi et al. 2013).

Due to the promising results, PACAP/VIP-based drug development has been initiated. One of these drugs is davenutide (NAP), an eight–amino acid molecule derived from activity-dependent neuroprotective protein (ADNP) (Belokopytov et al. 2011; Jehle et al. 2008). Its beneficial effects on retinopathy were studied in diabetic rats where the intraocular injection of NAP reduced apoptotic cell death via MAPK/ERK pathways (Scuderi et al. 2014), reduced HIF and VEGF levels (D’Amico et al. 2017a), and downregulated IL-1B (D’Amico et al. 2019). Similarly to PACAP, NAP improved the integrity of outer BRB and modulated the expression of apoptotic genes in ARPE-19 cells exposed to hyperglycemic-inflammatory or hyperglycemic-hypoxic insults (D’Amico et al. 2018, 2019).

We conclude that PACAP is part of the endogenous protective machinery, in lack of which retinopathies result in more severe disturbances.

## Data Availability

The data presented in this study are available on request from the corresponding author.

## References

[CR1] Allais A, Burel D, Isaac ER, Gray SL, Basille M, Ravni A, Sherwood NM, Vaudry H, Gonzalez BJ (2007). Altered cerebellar development in mice lacking pituitary adenylate cyclase-activating polypeptide. Eur J Neurosci.

[CR2] Armstrong BD, Abad C, Chhith S, Cheung-Lau G, Hajji OE, Nobuta H, Waschek JA (2008). Impaired nerve regeneration and enhanced neuroinflammatory response in mice lacking pituitary adenylyl cyclase activating peptide. Neuroscience.

[CR3] Atlasz T, Babai N, Kiss P, Reglodi D, Tamás A, Szabadfi K, Tóth G, Hegyi O, Lubics A, Gábriel R (2007). Pituitary adenylate cyclase activating polypeptide is protective in bilateral carotid occlusion-induced retinal lesion in rats. Gen Comp Endocrinol.

[CR4] Atlasz T, Szabadfi K, Kiss P, Marton Z, Griecs M, Hamza L, Gaal V, Biro Z, Tamas A, Hild G, Nyitrai M, Toth G, Reglodi D, Gabriel R (2011). Effects of PACAP in UV-A radiation-induced retinal degeneration models in rats. J Mol Neurosci.

[CR5] Atlasz T, Szabadfi K, Kiss P, Tamas A, Toth G, Reglodi D, Gabriel R (2010). Evaluation of the protective effects of PACAP with cell-specific markers in ischemia-induced retinal degeneration. Brain Res Bull.

[CR6] Atlasz T, Szabadfi K, Reglodi D, Kiss P, Tamas A, Toth G, Molnar A, Szabo K, Gabriel R (2009). Effects of pituitary adenylate cyclase activating polypeptide and its fragments on retinal degeneration induced by neonatal monosodium glutamate treatment. Ann N Y Acad Sci.

[CR7] Atlasz T, Vaczy A, Werling D, Kiss P, Tamas A, Kovacs K, Fabian E, Kvarik T, Mammel B, Danyadi B, Lokos E, Reglodi D (2016) Protective effects of PACAP in the retina. In: Reglodi D., Tamas A. (eds) Pituitary adenylate cyclase activating polypeptide — PACAP. Curr Top Neuroendocrinol vol 11. Springer, Cham

[CR8] Atlasz T, Werling D, Song S, Szabo E, Vaczy A, Kovari P, Tamas A, Reglodi D, Yu R (2019). Retinoprotective effects of TAT-bound vasoactive intestinal peptide and pituitary adenylate cyclase activating polypeptide. J Mol Neurosci.

[CR9] Belokopytov M, Shulman S, Dubinsky G, Gozes I, Belkin M, Rosner M (2011). Ameliorative effect of NAP on laser-induced retinal damage. Acta Ophthalmol.

[CR10] Biran J, Gliksberg M, Shirat I, Swaminathan A, Levitas-Djerbi T, Appelbaum L, Levkowitz G (2020). Splice-specific deficiency of the PTSD-associated gene PAC1 leads to a paradoxical age-dependent stress behavior. Sci Rep.

[CR11] Bonaventura G, Iemmolo R, D'Amico AG, La Cognata V, Costanzo E, Zappia M, D'Agata V, Conforti FL, Aronica E, Cavallaro S (2018). PACAP and PAC1R are differentially expressed in motor cortex of amyotrophic lateral sclerosis patients and support survival of iPSC-derived motor neurons. J Cell Physiol.

[CR12] Cavallaro G, Filippi L, Bagnoli P, La Marca G, Cristofori G, Raffaeli G, Padrini L, Araimo G, Fumagalli M, Groppo M, Dal Monte M, Osnaghi S, Fiorini P, Mosca F (2014). The pathophysiology of retinopathy of prematurity: an update of previous and recent knowledge. Acta Ophthalmol.

[CR13] Chen Y, Samal B, Hamelink CR, Xiang CC, Chen Y, Chen M, Vaudry D, Brownstein MJ, Hallenbeck JM, Eiden LE (2006). Neuroprotection by endogenous and exogenous PACAP following stroke. Regul Pept.

[CR14] Ciranna L, Costa L (2019). Pituitary adenylate cyclase-activating polypeptide modulates hippocampal synaptic transmission and plasticity: new therapeutic suggestions for Fragile X syndrome. Front Cell Neurosci.

[CR15] Connor KM, Krah NM, Dennison RJ, Aderman CM, Chen J, Guerin KI, Sapieha P, Stahl A, Willett KL, Smith LE (2009). Quantification of oxygen-induced retinopathy in the mouse: a model of vessel loss, vessel regrowth and pathological angiogenesis. Nat Protoc.

[CR16] D'Amico AG, Maugeri G, Bucolo C, Saccone S, Federico C, Cavallaro S, D'Agata V (2017). Nap interferes with hypoxia-inducible factors and VEGF expression in retina of diabetic rats. J Mol Neurosci.

[CR17] D'Amico AG, Maugeri G, Musumeci G, Reglodi D, D'Agata V (2021) PACAP and NAP: effect of two functionally related peptides in diabetic retinopathy. J Mol Neurosci in press 10.1007/s12031-020-01769-4 33403597

[CR18] D'Amico AG, Maugeri G, Rasà DM, Bucolo C, Saccone S, Federico C, Cavallaro S, D'Agata V (2017). Modulation of IL-1β and VEGF expression in rat diabetic retinopathy after PACAP administration. Peptides.

[CR19] D'Amico AG, Maugeri G, Rasà D, Federico C, Saccone S, Lazzara F, Fidilio A, Drago F, Bucolo C, D'Agata V (2019). NAP modulates hyperglycemic-inflammatory event of diabetic retina by counteracting outer blood retinal barrier damage. J Cell Physiol.

[CR20] D'Amico AG, Maugeri G, Rasà DM, La Cognata V, Saccone S, Federico C, Cavallaro S, D'Agata V (2018). NAP counteracts hyperglycemia/hypoxia induced retinal pigment epithelial barrier breakdown through modulation of HIFs and VEGF expression. J Cell Physiol.

[CR21] D’Amico AG, Maugeri G, Saccone S, Federico C, Cavallaro S, Reglodi D, D’Agata V (2020). PACAP modulates the autophagy process in an in vitro model of amyotrophic lateral sclerosis. Int J Mol Sci.

[CR22] D'Amico AG, Scuderi S, Saccone S, Castorina A, Drago F, D'Agata V (2013). Antiproliferative effects of PACAP and VIP in serum-starved glioma cells. J Mol Neurosci.

[CR23] Danyadi B, Szabadfi K, Reglodi D, Mihalik A, Danyadi T, Kovacs Z, Batai I, Tamas A, Kiss P, Toth G, Gabriel R (2014). PACAP application improves functional outcome of chronic retinal ischemic injury in rats – evidence from electroretinographic measurements. J Mol Neurosci.

[CR24] Di Y, Chen XL (2018). Inhibition of LY294002 in retinal neovascularization via down-regulation the PI3K/AKT-VEGF pathway in vivo and in vitro. Int J Ophthalmol.

[CR25] Endo K, Nakamachi T, Seki T, Kagami N, Wada Y, Nakamura K, Kishimoto K, Hori M, Tsuchikawa D, Shintani N, Hashimoto H, Baba A, Koide R, Shioda S (2011). Neuroprotective effect of PACAP against NMDA-induced retinal damage in the mouse. J Mol Neurosci.

[CR26] Farkas J, Sandor B, Tamas A, Kiss P, Hashimoto H, Nagy AD, Fulop BD, Juhasz T, Manavalan S, Reglodi D (2017). Early neurobehavioral development of mice lacking endogenous PACAP. J Mol Neurosci.

[CR27] Fulop DB, Humli V, Szepesy J, Ott V, Reglodi D, Gaszner B, Nemeth A, Szirmai A, Tamas L, Hashimoto H, Zelles T, Tamas A (2019). Hearing impairment and associated morphological changes in pituitary adenylate cyclase activating polypeptide (PACAP)-deficient mice. Sci Rep.

[CR28] Fulop BD, Sandor B, Szentleleky E, Karanyicz E, Reglodi D, Gaszner B, Zakany R, Hashimoto H, Juhasz T, Tamas A (2019). Altered notch signaling in developing molar teeth of pituitary adenylate cyclase-activating polypeptide (PACAP)-deficient mice. J Mol Neurosci.

[CR29] Gargiulo AT, Curtis GR, Barson JR (2020). Pleiotropic pituitary adenylate cyclase-activating polypeptide (PACAP): novel insights into the role of PACAP in eating and drug intake. Brain Res.

[CR30] Hashimoto H, Hashimoto R, Shintani N, Tanaka K, Yamamoto A, Hatanaka M, Guo X, Morita Y, Tanida M, Nagai K, Takeda M, Baba A (2009). Depression-like behavior in the forced swimming test in PACAP-deficient mice: amelioration by the atypical antipsychotic risperidone. J Neurochem.

[CR31] Hashimoto H, Shintani N, Tanaka K, Mori W, Hirose M, Matsuda T, Sakaue M, Miyazaki J, Niwa H, Tashiro F, Yamamoto K, Koga K, Tomimoto S, Kunugi A, Suetake S, Baba A (2001). Altered psychomotor behaviors in mice lacking pituitary adenylate cyclase-activating polypeptide (PACAP). Proc Natl Acad Sci U S A.

[CR32] Jehle T, Dimitriu C, Auer S, Knoth R, Vidal-Sanz M, Gozes I, Lagrèze WA (2008). The neuropeptide NAP provides neuroprotection against retinal ganglion cell damage after retinal ischemia and optic nerve crush. Graefes Arch Clin Exp Ophthalmol.

[CR33] Johnson GC, Parsons RL, May V, Hammack SE (2020). Pituitary adenylate cyclase-activating polypeptide-induced PAC1 receptor internalization and recruitment of MEK/ERK signaling enhance excitability of dentate gyrus granule cells. Am J Physiol Cell Physiol.

[CR34] Jozsa G, Fulop BD, Kovacs L, Czibere B, Szegeczki V, Kiss T, Hajdu T, Tamas A, Helyes Z, Zakany R, Reglodi D, Juhasz T (2019). Lack of pituitary adenylate cyclase-activating polypeptide (PACAP) disturbs callus formation. J Mol Neurosci.

[CR35] Jozsa G, Szegeczki V, Palfi A, Kiss T, Helyes Z, Fulop B, Cserhati C, Daroczi L, Tamas A, Zakany R, Reglodi D, Juhasz T (2018). Signalling alterations in bones of pituitary adenylate cyclase activating polypeptide (PACAP) gene deficient mice. Int J Mol Sci.

[CR36] Kawaguchi C, Isojima Y, Shintani N, Hatanaka M, Guo X, Okumura N, Nagai K, Hashimoto H, Baba A (2010). PACAP-deficient mice exhibit light parameter-dependent abnormalities on nonvisual photoreception and early activity onset. PLoS ONE.

[CR37] Kovacs-Valasek A, Szabadfi K, Denes V, Szalontai B, Tamas A, Kiss P, Szabo A, Setalo G, Reglodi D, Gabriel R (2017). Accelerated retinal aging in PACAP knock-out mice. Neuroscience.

[CR38] Kvarik T, Mammel B, Reglodi D, Kovacs K, Werling D, Bede B, Vaczy A, Fabian E, Toth G, Kiss P, Tamas A, Ertl T, Gyarmati J, Atlasz T (2016) PACAP is protective in the rat model of retinopathy of prematurity. J Mol Neurosci 60(2):179–185. 10.1007/s12031-016-0797-5 10.1007/s12031-016-0797-5 27561927

[CR39] Lauretta G, Ravalli S, Szychlinska MA, Castorina A, Maugeri G, D'Amico AG, D'Agata V, Musumeci G (2020). Current knowledge of pituitary adenylate cyclase activating polypeptide (PACAP) in articular cartilage. Histol Histopathol.

[CR40] Martínez-Rojas VA, Jiménez-Garduño AM, Michelatti D, Tosatto L, Marchioretto M, Arosio D, Basso M, Pennuto M, Musio C (2021). ClC-2-like chloride current alterations in a cell model of spinal and bulbar muscular atrophy, a polyglutamine disease. J Mol Neurosci.

[CR41] Maugeri G, D'Amico AG, Musumeci G, Reglodi D, D'Agata V (2020). Effects of PACAP on Schwann cells: focus on nerve injury. Int J Mol Sci.

[CR42] Maugeri G, D’Amico AG, Rasà DM, Saccone S, Federico C, Cavallaro S, D’Agata V (2018). PACAP and VIP regulate hypoxia-inducible factors in neuroblastoma cells exposed to hypoxia. Neuropeptides.

[CR43] Maugeri G, D’Amico AG, Reitano R, Magro G, Cavallaro S, Salomone S, D’Agata V (2016). PACAP and VIP inhibit the invasiveness of glioblastoma cells exposed to hypoxia through the regulation of HIFs and EGFR expression. Front Pharmacol.

[CR44] May V, Lutz E, MacKenzie C, Schutz KC, Dozark K, Braas KM (2010). Pituitary adenylate cyclase-activating polypeptide (PACAP)/PAC1HOP1 receptor activation coordinates multiple neurotrophic signaling pathways: Akt activation through phosphatidylinositol 3-kinase gamma and vesicle endocytosis for neuronal survival. J Biol Chem.

[CR45] Mori H, Nakamachi T, Ohtaki H, Yofu S, Sato A, Endo K, Iso Y, Suzuki H, Takeyama Y, Shintani N, Hashimoto H, Baba A, Shioda S (2010). Cardioprotective effect of endogenous pituitary adenylate cyclase-activating polypeptide on Doxorubicin-induced cardiomyopathy in mice. Circ J.

[CR46] Nakamachi T, Matkovits A, Seki T, Shioda S (2012). Distribution and protective function of pituitary adenylate cyclase-activating polypeptide in the retina. Front Endocrinol (Lausanne).

[CR47] Nakamachi T, Nakamura K, Oshida K, Kagami N, Mori H, Watanabe J, Arata S, Yofu S, Endo K, Wada Y, Hori M, Tsuchikawa D, Kato M, Shioda S (2011). Pituitary adenylate cyclase-activating polypeptide (PACAP) stimulates proliferation of reactive astrocytes in vitro. J Mol Neurosci.

[CR48] Nakamachi T, Ohtaki H, Seki T, Yofu S, Kagami N, Hashimoto H, Shintani N, Baba A, Mark L, Lanekoff I, Kiss P, Farkas J, Reglodi D, Shioda S (2016). PACAP suppresses dry eye signs by stimulating tear secretion. Nat Commun.

[CR49] Nakamachi T, Ohtaki H, Yofu S, Dohi K, Watanabe J, Mori H, Sato A, Hashimoto H, Shintani N, Baba A, Shioda S (2010). Endogenous pituitary adenylate cyclase activating polypeptide is involved in suppression of edema in the ischemic brain. Acta Neurochir Suppl.

[CR50] Nonaka N, Banks WA, Shioda S (2020). Pituitary adenylate cyclase-activating polypeptide: Protective effects in stroke and dementia. Peptides.

[CR51] Ohtaki H, Nakamachi T, Dohi K, Aizawa Y, Takaki A, Hodoyama K, Yofu S, Hashimoto H, Shintani N, Baba A, Kopf M, Iwakura Y, Matsuda K, Arimura A, Shioda S (2006). Pituitary adenylate cyclase-activating polypeptide (PACAP) decreases ischemic neuronal cell death in association with IL-6. Proc Natl Acad Sci U S A.

[CR52] Ohtaki H, Satoh A, Nakamachi T, Yofu S, Dohi K, Mori H, Ohara K, Miyamoto K, Hashimoto H, Shintani N, Baba A, Matsunaga M, Shioda S (2010). Regulation of oxidative stress by pituitary adenylate cyclase-activating polypeptide (PACAP) mediated by PACAP receptor. J Mol Neurosci.

[CR53] Racz B, Gallyas F, Kiss P, Tamas A, Lubics A, Lengvari I, Roth E, Toth G, Hegyi O, Verzar Z, Fabricsek C, Reglodi D (2007). Effects of pituitary adenylate cyclase activating polypeptide (PACAP) on the PKA-Bad-14–3-3 signaling pathway in glutamate-induced retinal injury in neonatal rats. Neurotox Res.

[CR54] Racz B, Gallyas F, Kiss P, Toth G, Hegyi O, Gasz B, Borsiczky B, Ferencz A, Roth E, Tamas A, Lengvari I, Lubics A, Reglodi D (2006). The neuroprotective effects of PACAP in monosodium glutamate-induced retinal lesion involves inhibition of pro-apoptotic signaling pathways. Regul Pept.

[CR55] Racz B, Gasz B, Gallyas F, Kiss P, Tamas A, Szanto Z, Lubics A, Lengvari I, Toth G, Hegyi O, Roth E, Reglodi D (2008). PKA-Bad-14-3-3 Akt-Bad-14-3-3 signaling pathways are involved in the protective effects of PACAP against ischemia/reperfusion-induced cardiomyocyte apoptosis. Regul Pept.

[CR56] Reglodi D, Atlasz T, Szabo E, Jungling A, Tamas A, Juhasz T, Fulop BD, Bardosi A (2018). PACAP deficiency as a model of aging. Geroscience.

[CR57] Reglodi D, Jungling A, Longuespée R, Kriegsmann J, Casadonte R, Kriegsmann M, Juhasz T, Bardosi S, Tamas A, Fulop BD, Kovacs K, Nagy Z, Sparks J, Miseta A, Mazzucchelli G, Hashimoto H, Bardosi A (2018). Accelerated pre-senile systemic amyloidosis in PACAP knock-out mice - a protective role of PACAP in age-related degenerative processes. J Pathol.

[CR58] Reglodi D, Kiss P, Horvath G, Lubics A, Laszlo E, Tamas A, Racz B, Szakaly P (2012). Effects of pituitary adenylate cyclase activating polypeptide in the urinary system, with special emphasis on its protective effects in the kidney. Neuropeptides.

[CR59] Riedel CS, Georg B, Fahrenkrug J, Hannibal J (2020). Altered light induced EGR1 expression in the SCN of PACAP deficient mice. PLoS ONE.

[CR60] Rivnyak A, Kiss P, Tamas A, Balogh D, Reglodi D (2018). Review on PACAP-induced transcriptomic and proteomic changes in neuronal development and repair. Int J Mol Sci.

[CR61] Scuderi S, D'Amico AG, Castorina A, Federico C, Marrazzo G, Drago F, Bucolo C, D'Agata V (2014). Davunetide (NAP) protects the retina against early diabetic injury by reducing apoptotic death. J Mol Neurosci.

[CR62] Scuderi S, D'Amico AG, Castorina A, Imbesi R, Carnazza ML, D'Agata V (2013). Ameliorative effect of PACAP and VIP against increased permeability in a model of outer blood retinal barrier dysfunction. Peptides.

[CR63] Soles-Tarres I, Cabezas-Llobet N, Vaudry D, Xifro X (2020). Protective effects of pituitary adenylate cyclase-activating polypeptide and vasoactive intestinal peptide against cognitive decline in neurodegenerative diseases. Front Cell Neurosci.

[CR64] Szabadfi K, Atlasz T, Kiss P, Danyadi B, Tamas A, Helyes Z, Hashimoto H, Shintani N, Baba A, Toth G, Gabriel R, Reglodi D (2012). Mice deficient in pituitary adenylate cyclase activating polypeptide (PACAP) are more susceptible to retinal ischemic injury in vivo. Neurotox Res.

[CR65] Szabo A, Danyadi B, Bognar E, Szabadfi K, Fabian E, Kiss P, Mester L, Manavalan S, Atlasz T, Gabriel R, Toth G, Tamas A, Reglodi D, Kovacs K (2012). Effect of PACAP on MAP kinases, Akt and cytokine expressions in rat retinal hypoperfusion. Neurosci Lett.

[CR66] Szegeczki V, Bauer B, Jungling A, Fulop BD, Vago J, Perenyi H, Tarantini S, Tamas A, Zakany R, Reglodi D, Juhasz T (2019) Age-related alterations of articular cartilage in pituitary adenylate cyclase-activating polypeptide (PACAP) gene-deficient mice. Geroscience 41(6):775-793. 10.1007/s11357-019-00097-9 10.1007/s11357-019-00097-9 PMC6925077 31655957

[CR67] Tamas A, Gabriel R, Racz B, Denes V, Kiss P, Lubics A, Lengvari I, Reglodi D (2004). Effects of pituitary adenylate cyclase activating polypeptide in retinal degeneration induced by monosodium-glutamate. Neurosci Lett.

[CR68] Tamas A, Szabadfi K, Nemeth A, Fulop B, Kiss P, Atlasz T, Gabriel R, Hashimoto H, Baba A, Shintani N, Helyes Z, Reglodi D (2012). Comparative examination of inner ear in wild type and pituitary adenylate cyclase activating polypeptide (PACAP)-deficient mice. Neurotox Res.

[CR69] Tan YV, Abad C, Lopez R, Dong H, Liu S, Lee A, Gomariz RP, Leceta J, Waschek JA (2009). Pituitary adenylyl cyclase-activating polypeptide is an intrinsic regulator of Treg abundance and protects against experimental autoimmune encephalomyelitis. Proc Natl Acad Sci U S A.

[CR70] Toth D, Szabo E, Tamas A, Juhasz T, Horvath G, Fabian E, Opper B, Szabo D, Maugeri G, D'Amico AG, D'Agata V, Vicena V, Reglodi D (2020). Protective effects of PACAP in peripheral organs. Front Endocrinol (Lausanne).

[CR71] Tsuchikawa D, Nakamachi T, Tsuchida M, Wada Y, Hori M, Farkas J, Yoshikawa A, Kagami N, Imai N, Shintani N, Hashimoto H, Atsumi T, Shioda S (2012). Neuroprotective effect of endogenous pituitary adenylate cyclase-activating polypeptide on spinal cord injury. J Mol Neurosci.

[CR72] Vaczy A, Kovari P, Kovacs K, Farkas K, Szabo E, Kvarik T, Kocsis B, Fulop B, Atlasz T, Reglodi D (2018). Protective role of endogenous PACAP in inflammation-induced retinal degeneration. Curr Pharm Des.

[CR73] Vaudry D, Hamelink C, Damadzic R, Eskay RL, Gonzalez B, Eiden LE (2005). Endogenous PACAP acts as a stress response peptide to protect cerebellar neurons from ethanol or oxidative insult. Peptides.

[CR74] Vincze A, Reglodi D, Helyes Z, Hashimoto H, Shintani N, Abraham H (2011). Role of endogenous pituitary adenylate cyclase activating polypeptide (PACAP) in myelination of the rodent brain: lessons from PACAP-deficient mice. Int J Dev Neurosci.

[CR75] Werling D, Reglodi D, Banks WA, Salameh TS, Kovacs K, Kvarik T, Vaczy A, Kovacs L, Mayer F, Danyadi B, Lokos E, Tamas A, Toth G, Biro Z, Atlasz T (2016). Ocular delivery of PACAP1-27 protects the retina from ischemic damage in rodents. Invest Ophthalmol Vis Sci.

[CR76] Werling D, Banks WA, Salameh TS, Kvarik T, Kovacs LA, Vaczy A, Szabo E, Mayer F, Varga R, Tamas A, Toth G, Biro Z, Atlasz T, Reglodi D (2017). Passage through the ocular barriers and beneficial effects in retinal ischemia of tpical application of PACAP1-38 in rodents. Int J Mol Sci.

[CR77] Yamada K, Matsuzaki S, Hattori T, Kuwahara R, Taniguchi M, Hashimoto H, Shintani N, Baba A, Kumamoto N, Yamada K, Yoshikawa T, Katayama T, Tohyama M (2010). Increased stathmin1 expression in the dentate gyrus of mice causes abnormal axonal arborizations. PLoS ONE.

